# Sertoli Cell-Germ Cell Interactions Within the Niche: Paracrine and Juxtacrine Molecular Communications

**DOI:** 10.3389/fendo.2022.897062

**Published:** 2022-06-10

**Authors:** Marie-Claude Hofmann, Elena McBeath

**Affiliations:** Department of Endocrine Neoplasia and Hormonal Disorders, MD Anderson Cancer Center, Houston, TX, United States

**Keywords:** Sertoli cell, germ cell, growth factors, self-renewal, differentiation

## Abstract

Male germ cell development depends on multiple biological events that combine epigenetic reprogramming, cell cycle regulation, and cell migration in a spatio-temporal manner. Sertoli cells are a crucial component of the spermatogonial stem cell niche and provide essential growth factors and chemokines to developing germ cells. This review focuses mainly on the activation of master regulators of the niche in Sertoli cells and their targets, as well as on novel molecular mechanisms underlying the regulation of growth and differentiation factors such as GDNF and retinoic acid by NOTCH signaling and other pathways.

## Introduction

### The Niche Microenvironment

Maintenance, repair, and regeneration of many mammalian organs depend on adult stem cells. Stem cells proliferate and differentiate to replace mature functional cells within tissues that have either high turnover such as blood, testis, and epithelia (intestine, skin, and respiratory tract), or tissues that have low turnover but a high regenerative potential upon disease or injury such as liver, pancreas, skeletal muscle, and bone (1). Precise regulation of adult stem cell fate is therefore critical for the support of tissue homeostasis, and stem cell maintenance must involve a fine balance between genetic and epigenetic mechanisms, external factors from the microenvironment and systemic support, and multiple signaling pathways elicited by paracrine and juxtacrine factors.

Over the years, evidence has accumulated showing that stem cell self-renewal depends on the constituents of their microenvironment called the niche (2, 3) and that in turn stem cells influence their own environment (4–6). The constituents of the niche can be classified into adjacent supporting cells such as fibroblasts, tissue macrophages, glial cells (brain), osteoblasts (bone marrow), Sertoli cells (testis) and myofibroblasts (gut), together with paracrine and juxtacrine factors secreted by these supporting cells, and the extracellular matrix. Once they leave the niche, stem cells become progenitor cells that are less plastic and differentiate at the expense of their immortality. Over the last 15 years, critical cellular and molecular components of the specialized niche microenvironment have begun to be unveiled in several tissues. Advanced techniques in lineage-tracing, endogenous cell and gene/protein deletions in animal models, and high-resolution microscopy have significantly improved our understanding of the molecular and cellular intricacies that maintain and integrate the many activities required to sustain tissue homeostasis.

### The Spermatogonial Stem Cell Niche

In the mammalian testis, the male germline produces a life-long supply of haploid spermatozoa through the highly regulated and coordinated process of spermatogenesis. This process starts with the self-renewal of a small pool of diploid stem cells called spermatogonial stem cells (SSCs or A_single_ spermatogonia), which can self-renew to maintain the pool or give rise to more mature germ cells called A_paired_ and A_aligned_ spermatogonia. Collectively, A_single_, A_paired_ and A_aligned_ spermatogonia are called undifferentiated spermatogonia (7). These cells further differentiate into differentiating spermatogonia and spermatocytes that undergo meiosis, producing haploid spermatids that will mature into spermatozoa. The longevity and the high output of sperm cell production relies therefore primarily on the proper maintenance of the pool of SSCs and their proliferation. Like other types of stem cells, SSCs rely on their micro-environment to sustain their growth and to initiate differentiation that signals their release from the basal part of the seminiferous epithelium and exit from the niche.

**Figure 1:**
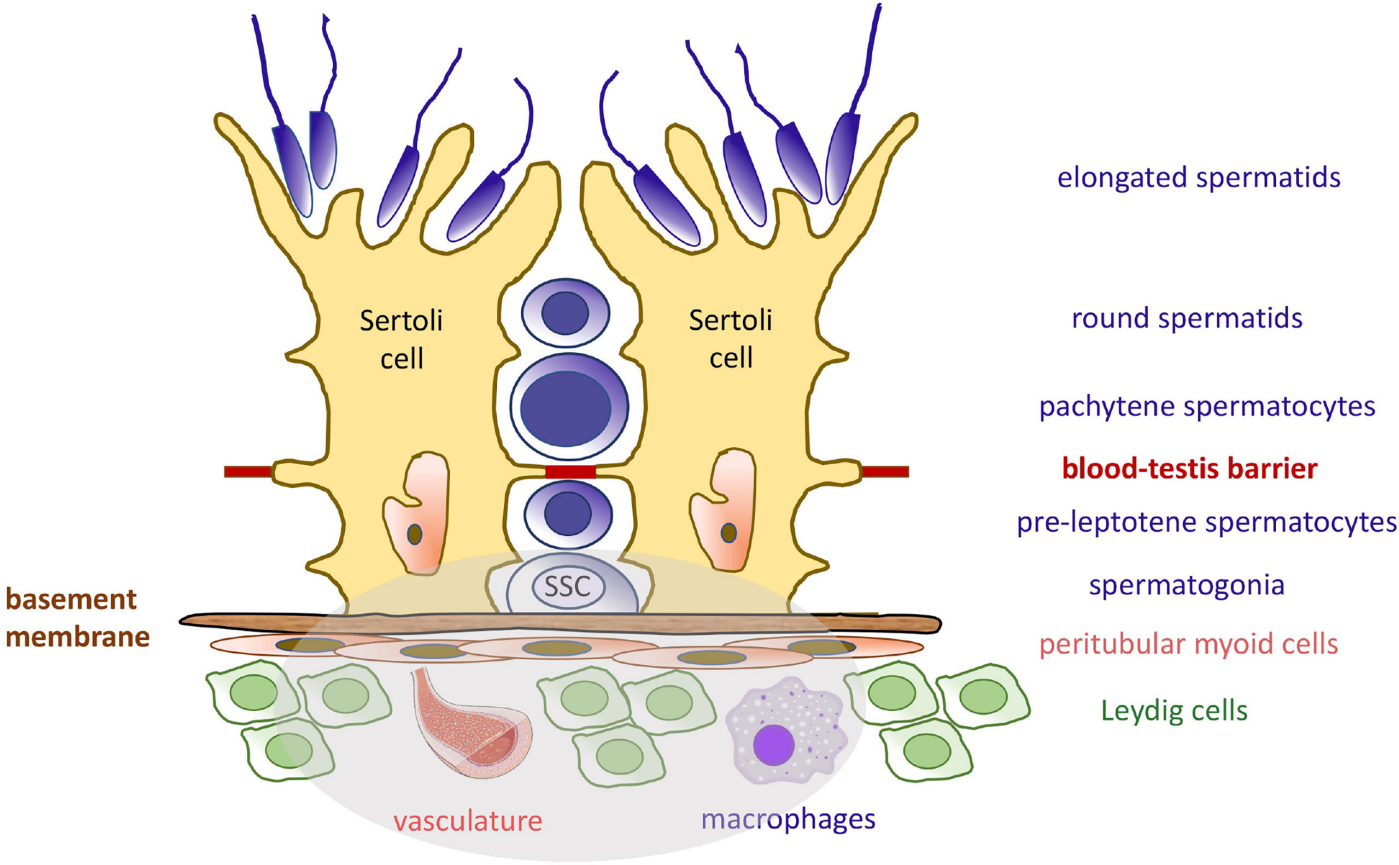
Seminiferous Epithelium Organization and the Spermatogonial Stem Cell Niche. The seminiferous epithelium consists of germ cells (blue) and the somatic Sertoli cells (yellow). Sertoli cells produce many factors needed at various developmental steps during the spermatogenic process. The blood-testis barrier separates diploid germ cells from more mature cells and provide an immuno-privileged microenvironment for the completion of meiosis. Like Sertoli cells, the spermatogonial stem cells (SSCs) are attached to the basement membrane. They rely on specific growth factors for self-renewal and maintenance of the pool. These molecules are produced by Sertoli cells, peritubular myoid cells, Leydig cells, and macrophages, as well as the vasculature. The components of the SSC niche are highlighted in the grey area.

SSCs reside on the basement membrane that supports the seminiferous epithelium. They are in intimate physical contact with highly specialized somatic niche cells, the Sertoli cells, which directly provide soluble growth factors and membrane-bound signals to the germ cells (8). Other niche cell types have been recently investigated, including peritubular myoid cells, interstitial cells (macrophages and Leydig cells), and endothelial cells from the vascular network, which all produce critical growth factors (**Figure 1**) (9–15). Because of their direct physical association with germ cells, their secretion of growth factors and basement membrane components, and their architectural support of the seminiferous epithelium, Sertoli cells are considered the most important contributor of the testicular niche, and the regulation of their molecular communications with SSCs and more mature premeiotic germ cells will be the subject of this review.

## Sertoli Cells as Structural Niche Organizers

It is now established that the number of Sertoli cells increases during fetal development due to growth stimulation through FSH/FSHR signaling. Sertoli cells proliferate up to day 15 after birth in mice and 17 days after birth in rats, after which the number of Sertoli cells reaches its peak and remain constant throughout life unless altered by insult and aging. Therefore, the number of Sertoli cells is finite and its maintenance is crucial for life-long spermatogenesis. Several years ago, de Franca et al. induced experimental hypothyroidism in the rat with propylthiouracil (PTU) administrated neonatally. The treatment significantly increased the period of Sertoli cell proliferation and therefore increased their number at puberty and beyond. This also increased germ cell number and the size of the testes (16). However, direct evidence that Sertoli cells indeed provide a structural and functional SSC niche support was provided by Oatley and colleagues (17). The authors treated male mouse pups with PTU, which led to increased Sertoli cell and germ cell numbers in the adult testes. Next, by using these mice as germ cells recipients after busulfan treatment destroyed their endogenous germ cells, they showed a significant increase of colonization by normal donor SSCs after transplantation. This demonstrated an increased presence of functional niches. Because neither the vasculature nor interstitial cell populations were altered in the PTU recipient model, they concluded that Sertoli cells are the most critical somatic cell type in the testis and that they create the SSC niche.

## Master Regulators of the Niche

The germ cell and Sertoli cell behaviors leading to the establishment of the spermatogenic stem cell niche in the early postnatal testis are well known. In addition to Sertoli cell proliferation leading to the expansion of the niche units until puberty, one of the most striking cellular behavior is the movement of pro-spermatogonia, or gonocytes, toward the periphery of the cords at around day 3-4 after birth in rodents, and 8-12 weeks after birth in humans (18, 19). By postnatal day 6 in the mouse, about 90% of pro-spermatogonia have reached the basal lamina, have become SSCs and rapidly differentiate (20), whereas germ cells that failed to migrate have died (21). The past fifteen years have seen a growing interest in understanding how these processes are regulated and the discovery of Sertoli cell-specific genes that are master determinants of the niche has become a priority.

DMRT1 (*Doublesex* and *Mab-3 *related transcription factor 1) is a conserved gene that* *is expressed in the testes of all vertebrates. In the mouse, DMRT1 expression starts at the genital ridge stage and continues throughout adult life. DMRT1 is required for normal sexual development, and defective expression leads to abnormal testicular formation and XY feminization (22). While both germ cells and Sertoli cells express the gene, Sertoli cell-specific knockout of *Dmrt1* led to testicular abnormalities at around day 7 post-partum (22–25). Sertoli cells lacking DMRT1 re-expressed Forkhead box L2 (FOXL2), a female gonad determinant (26). The cells could not polarize, reprogrammed into granulosa cells, and seminiferous tubule lumens did not form (22). Consequently, SSCs and undifferentiated spermatogonia were not maintained at the tubule periphery, the germ cell population remained disorganized, and germ cells died after meiotic arrest. This indicated that DMRT1 antagonizes FOXL2 and functions as a repressor of the female gonad development. Further, DMRT1 is also a known activator of androgen receptor (AR) (27, 28) and is crucial for cellular junction formation and function by driving the expression of *Claudin 11 (Cldn11), Vinculin (Vcl)*, and *gap junction protein alpha 3 (Gja3)* (**Table 1**), therefore controlling the structural niche as well (28, 48, 79, 120).

**Table 1:** Names and functions of proteins discussed in this review.

Protein	UniProt ID (mouse, unless specified)	Cell Type	Function in the testis	References
ACTB	P60710	Sertoli cells	**Beta-Actin**. Component of adherens junctions.	29, 30
AIP1 (WDR1)	P60710	Sertoli cells	**Actin-Interacting Protein 1**. Functions as Actin disassembly factor, promotes germ cell movement toward the basement membrane.	31
AIP1 (WDR1)	P60710	Pro-spermatogonia/Undifferentiated spermatogonia	**Actin-Interacting Protein 1**. Functions as Actin disassembly factor, promotes germ cell movement toward the basement membrane.	31
AMH	P27106	Sertoli cells, immature	**Anti-Mullerian Hormone**. Regression of Müllerian ducts in male fetuses.	32, 33
AR (NR3C4)	P19091	Sertoli cells	**Androgen receptor**. Responsible for binding of Testosterone/Dihydrotestosterone.	27, 28, 34
ARID4A/ARI4A	F8VPQ2	Sertoli cells	**AT-Rich Interaction Domain 4A.** Maintains the blood-testis barrier. Knock-out induces meiotic arrest.	33, 35
ARID4B/ARI4B	A2CG63	Sertoli cells	**AT-Rich Interaction Domain 4B**. Supports the SSC niche. Transcriptional coactivator for AR.	33, 34, 36
BCL6B	O88282	Spermatogonial stem cells	**B-Cell CLL/Lymphoma 6, Member B**. Supports self-renewal.	37, 38
BEX1	Q9HBH7 (human)	Human Sertoli cells, Stage b (8-11 year old)	**Brain Expressed X-Linked Protein 1**. Transcription regulator. Plays a role in cell cycle progression in Stage b human Sertoli cells.	30
CCL3	P10855	Sertoli cells, perinatal	**C-C Motif Chemokine Ligand 3**. Guides pro-spermatogonia toward the basement membrane.	39–41
CCL9	P51670	Sertoli cells, perinatal	**C-C Motif Chemokine Ligand 9**. Guides pro-spermatogonia toward the basement membrane. Maintains SSCs within the niche.	42
CCR1	P51675	Pro-spermatogonia, undifferentiated spermatogonia	**C-C Motif Chemokine Receptor 1**. Receptor for CCL3 and CCL9.	39
CDC42	P60766	Sertoli cells	**Cell Division Cycle Protein 42**. Involved in cell polarity and migration. Regulation of the blood-testis barrier and Sertoli cell polarity.	43–47
CDH1	P09803	Sertoli cells	**E-cadherin/cadherin-1**. Calcium-dependent cell adhesion protein.	29
CLDN11/ CLD11	Q60771	Sertoli cells	**Claudin 11**. Tight junction protein at the blood-testis barrier.	28, 48
CSF1	P07141	Leydig cells	**Macrophage Colony Stimulating Factor 1**. Enhances self-renewal of spermatogonial stem cells.	12
CST9L	Q9H4G1 (human)	Human Sertoli cells, Stage c (17 year old to adult)	**Cystatin 9 Like**. Tissue remodeling during early testis development. Also present in adult Sertoli cells.	30, 49
CTNNB1	Q02248	Spermatocytes and spermatids	**Catenin Beta 1**. Maintenance of post-mitotic germ cells.	50–52
CXCL12/SDF1	P40224	Sertoli cells	**C-X-C Motif Chemokine Ligand 12.** Guides pro-spermatogonia toward the basement membrane. Maintains SSCs within the niche.	41 53
CXCR4	P70658	Pro-spermatogonia, undifferentiated spermatogonia	**C-X-C Motif Chemokine Receptor 4**. Receptor for CXCL12.	40
CYP26B1	Q811W2	Sertoli cells, immature and postnatal	**Cytochrome P450 Family 26 Subfamily B Member 1**. Inactivates retinoic acid through oxidation.	54–56
DEFB119	Q8N690 (human)	Human Sertoli cells, Stage c (17 year old to adult)	**Defensin Beta 119**. Anti-microbial defense in the male reproductive tract.	30, 57
DMRT1	Q9QZ59	Sertoli cells, immature and adult	**Doublesex And Mab-3 Related Transcription Factor 1.** Required for normal testis development and maintenance. Antagonist of FOXL2.	22, 23, 28, 58
DMRT1	Q9QZ59	Germ cells	**Doublesex And Mab-3 Related Transcription Factor 1**. Required for SSC maintenance and germ cell mitosis/meiosis decision.	24, 25
EGF	P01133 (human)	Human Sertoli cells, Stage a (2-5 year old)	**Epidermal Growth Factor.** Produced by Sertoli cells. Germ cell maintenance/proliferation.	30, 59
EGR3	Q06889 (human)	Human Sertoli cells, Stage a (2-5 year old)	**Early Growth Response 3**. Induced by mitogenic stimulation of Sertoli cells.	30
ENO1/ENOA	P06733 (human)	Human Sertoli cells, Stage b (8-11 year old)	**Enolase 1**. Growth control, cell metabolism.	30
ERK5/MAPK7	Q13164 (human)	Human Sertoli cells, Stage a (2-5 year old)	**Mitogen-Activated Protein Kinase 7.** Proliferation, differentiation, transcription regulation and development of Sertoli cells.	30
ETV5	Q9CXC9	Sertoli cells	**ETS Variant Transcription Factor 5**. Induces the production of chemokines and maintains SSC homing within the niche	42, 60, 61
ETV5	Q9CXC9	Spermatogonial stem cells	**ETS Variant Transcription Factor 5**. Induces the production of CXCR4 and Brachyury (T) and maintains SSC homing within the niche.	62 63
FGF2	P15655	Sertoli cells	**Fibroblast Growth Factor 2**. SSC self-renewal.	38, 64–68
FOXL2	O88470	Granulosa cells	**Forkhead Box L2**. Ovarian development and function. Repression of somatic testis determination. Antagonist of DMRT1.	22, 26
FSH	Q60687	Anterior pituitary cells	**Follicle Stimulating Hormone Subunit Beta**. Induces Sertoli cell proliferation in early development. Induces Sertoli cells to secrete androgen-binding proteins (ABPs), and stimulates inhibin B secretion.	69, 70
FSHR	P35378	Sertoli cells	**Follicle Stimulating Hormone Receptor**	71
GATA4	Q08369	Sertoli cells	**GATA Binding Protein 4**. Embryonic testis development, Sertoli cell maintenance, production of chemokines, SSC niche maintenance.	41, 58, 72–74
GDNF	P48540	Sertoli cells, postnatal	**Glial Cell Derived Neurotrophic Factor**. SSC self-renewal	66, 75;
GDNF	P48540	Sertoli cells, prenatal	**Glial Cell Derived Neurotrophic Factor.** Pro-spermatogonia maintenance.	76
GFRA1	P97785	Undifferentiated spermatogonia	**GDNF Family Receptor Alpha 1**. Co-receptor of RET	77, 78
GJA3 (CX46)	Q64448	Sertoli cells	**Gap Junction Protein Alpha 3**. Connexin 46. Gap Junction Protein, component of the blood-testis barrier.	28, 79
HES1	P35428	Sertoli cells	**HES Family BHLH Transcription Factor 1.** Target/mediator of NOTCH signaling. Inhibits GDNF and CYP26B1 expression.	56, 80
HEY1	Q9WV93	Sertoli cells	**Hes Related Family BHLH Transcription Factor With YRPW Motif 1.** Target/mediator of NOTCH signaling. Inhibits GDNF and CYP26B1 expression.	56, 80
HOPX	Q9BPY8 (human)	Human Sertoli cells, Stage c (17 year old to adult)	**HOP Homeobox**. Growth suppression and differentiation.	30, 81
IGF1	P05019 (human)	Human Sertoli cells, Stage a (2-5 year old)	**Insulin-Like Growth Factor 1**. Produced by Sertoli cells. Germ cell proliferation.	30, 82
INHBB	Q04999	Sertoli cell	**Inhibin Subunit Beta B**. Testis development. Marker of Sertoli cells function and germ cell numbers. Regulation of FSH secretion by pituitary.	33, 83, 84
JAG1	Q9QXX0	Undifferentiated spermatogonia	**Jagged 1**. Canonical NOTCH ligand.	55, 85
JUN	P05627	Sertoli cell	**Jun Proto-Oncogene**. AP-1 transcription factor complex subunit. Sertoli cell function, maintenance of the blood-testis barrier.	30, 86
KIT	P05532	Differentiating spermatogonia	**KIT Proto-Oncogene, Receptor Tyrosine Kinase.** Proliferation and differentiation.	87–89
KIT	P05532	Primordial germ cells	**KIT Proto-Oncogene, Receptor Tyrosine Kinase.** Proliferation and Survival.	90
KIT	P10721 (human)	Seminoma cells	**KIT Proto-Oncogene, Receptor Tyrosine Kinase.** Mutated and constitutively activated in 25% of seminoma.	91
KITL	P20826	Sertoli cell	**KIT Ligand.** Proliferation and differentiation of germ cells.	89, 92–95
LIF	P42703	Sertoli cell	**Leukemia Inhibitory Factor**. Maintenance of spermatogonial stem cell survival.	10, 66, 96
LIN28	Q8K3Y3	Pro-spermatogonia, undifferentiated spermatogonia	**Lin-28 Homolog A**. Pluripotency and SSC self-renewal.	97, 98
NFKB1	P25799	Sertoli cell	**Nuclear Factor Kappa B1**. Pleiotropic transcription factor.	99
NOTCH1	Q01705	Sertoli cell	**NOTCH Receptor 1**. Intercellular signaling pathway regulating cell fate specification and differentiation	56, 80, 85, 100
NR3C1	P06537	Fetal and perinatal Sertoli cell	**Nuclear Receptor Subfamily 3 Group C Member 1**. Glucocorticoid receptor. Possible link between stress and testicular function.	33, 101, 102;
NR3C1	P06537	Germ cell (spermatogonia)	**Nuclear Receptor Subfamily 3 Group C Member 1**. Glucocorticoid receptor. Possible link between stress and testicular function.	101
NR4A1	P22736 (human)	Human Sertoli cells, Stage a (2-5 year old)	**Nuclear receptor subfamily 4 group A member 1**. Proliferation, chemotaxis.	30
PAK1	O88643	Sertoli cell	**P21 Protein (Cdc42/Rac)-Activated Kinas**e 1. Canonical target of RHO GTPases.	44
PDGFA	P20033	Sertoli cells, perinatal	**Platelet-derived growth factor subunit A.** Germ cell proliferation.	103–106
PDGFB	P31240	Sertoli cells, perinatal	**Platelet-derived growth factor subunit A.** Germ cell proliferation.	103–106
PLZF (ZBTB16)	A3KMN0	Undifferentiated spermatogonia	**Zinc Finger And BTB Domain Containing 16**. Represses KIT in undifferentiated spermatogonia.	107, 108
RAC1	P63001	Sertoli cell	**Ras-related C3 botulinum toxin substrate 1.** Sertoli cell polarity.	109
RARA/G	P18911	Germ cells, undifferentiated	**Retinoic acid receptor alpha/gamma.** Germ cell differentiation.	68
RBPJ	P31266	Sertoli cells	**Immunoglobulin Kappa J Region Recombination Signal Binding Protein 1.** Transcription factor, mediator of all activated NOTCH receptors	80, 100, 100
RET	P35546	Germ cell, undifferentiated	**Ret Proto-Oncogene, Rearranged During Transfection**. SSC self-renewal, undifferentiated spermatogonia proliferation.	77, 78;
RET	P35546	Germ cell, fetal	**Ret Proto-Oncogene, Rearranged During Transfection**. Maintenance of fetal germ cells.	110
RHOA	P61586 (human)	Human Sertoli cells, Stage b (8-11 year old)	**Transforming protein RhoA.** Sertoli cell polarity, junction remodelling	30, 111
RHOX5	P52651	Sertoli cells	**Homeobox protein Rhox5.** Regulation of germ cell apoptosis.	34, 112,
S100A13	Q99584 (human)	Human Sertoli cells, Stage b (8-11 year old)	**S100 Calcium Binding Protein A13**. Cell cycle progression and differentiation.	30
SIN3A	Q60520	Sertoli cell	**Switch-insensitive 3a (SIN3A).** Co-repressor, regulation of chemokines expression.	113, 114
SOHlH1	Q6IUP1	Differentiating spermatogonia	**Spermatogenesis- and oogenesis-specific basic helix-loop-helix-containing protein 1.** Upregulation of KIT receptor expression.	115;
SOHlH2	Q9D489	Differentiating spermatogonia	**Spermatogenesis- and oogenesis-specific basic helix-loop-helix-containing protein 1**. Upregulation of KIT receptor expression.	115;
SOX9	Q04887	Sertoli cells	**SRY-Box Transcription Factor 9**. Sex determination. Maintenance of Sertoli cell functions.	58, 61, 116
VEGFA	Q00731	Sertoli cells, perinatal	**Vascular endothelial growth factor A**. Maintenance of spermatogonial stem cells.	117, 118
VEGFA	Q00731	Germ cells, perinatal	**Vascular endothelial growth factor A**. Maintenance of spermatogonial stem cells.	117
VEGFA	Q00731	Interstitial cells	**Vascular endothelial growth factor A**. Maintenance of spermatogonial stem cells.	117
VEGFA164	Q00731	Sertoli cells	**Vascular endothelial growth factor A, VEGFA164 isoform.** SSC self-renewal.	119
VCL	Q64727	Sertoli cells	**Vinculin**. Actin filament (F-actin)-binding protein. Cell-cell adhesion, adherens junction, ectoplasmic specializtion.	28, 120
WNT5A	P22725	Sertoli cells	**Wingless-Type MMTV Integration Site Family, Member 5A**. SSC maintenance and survival. CTNNB1 independent.	50, 121
WNT3A	P27467	Sertoli cells	**Wingless-Type MMTV Integration Site Family, Member 5A**. Proliferation of progenitor spermatogonia exiting the SSC state. CTNNB1-dependent.	122
WT1	P22561	Sertoli cells, fetal and adult	**Wilms tumor protein homolog 1.** Testis development, lineage maintenance of Sertoli cells.	27, 33
WTAP	Q9ER69	Sertoli cell	**Wilms tumor protein homolog 1-associated protein**. Mediates N6-methyladenosine (m6A) methylation of RNAs.	33, 123

In 2015, Chen and colleagues demonstrated that targeted loss of *Gata4*, a known Sertoli cell marker also involved in mouse genital ridge initiation, sex determination, and embryonic testis development (72–74), resulted in a loss of the establishment and maintenance of the SSC pool, and led to Sertoli cell-only syndrome (41). Loss of *Gata4* altered the expression of a number of chemokines, including *Cxcl12* (SFD1, binding to the CXCR4 receptor) and *Ccl3* (binding to the CCR1 receptor), which are known to guide pro-spermatogonia toward the basement membrane and the niche provided by Sertoli cells (39, 40). Similarly, another Sertoli cell transcription factor, ETV5, was found to directly bind to the promoter of the chemokine *Ccl9*. CCL9 facilitated chemoattraction of stem/progenitor spermatogonia, which express CCR1, the receptor for CCL9 (42) (**Table 1**). Together, these results revealed a novel role for GATA4 and ETV5 in organizing the SSC niche *via* the transcriptional regulation of chemokine signaling shortly after birth. More recently, Alankarage and colleagues demonstrated that *Etv5* in Sertoli cells is directly under control of SOX9, a transcription factor that specifies the function of Sertoli cells and their differentiation from somatic cell precursors (61).

Migration of pro-spermatogonia to the basement membrane and niches provided by Sertoli cells is also dependent on AIP1, a β-actin-interacting protein that mediates β-actin (ACTB) disassembly (29, 31). Sertoli and germ cell-specific deletion of mouse *Aip1* each led to significant defects in germ cell migration at postnatal day 4, which corresponded to elevated numbers of actin filaments in the affected cells. Increased actin filaments might have caused cytoskeletal changes that impaired E-cadherin (CDH1) regulation in Sertoli cells and germ cells, decreasing germ cell motility. *Aip1* deletion in Sertoli cells did not affect the expression and secretion of growth factors, suggesting that the disruption of SSC migration and function results from architectural changes in the postnatal niche.

Another determinant of the perinatal niche, CDC42, was recently identified by Mori et al. (46). Together with RAC1 and RHOA, CDC42 is a member of the RHO family of small GTP-ases, which are mainly involved in cell polarity and migration (43, 111). Importantly, a possible role of the small GTP-ases CDC42 and RAC1 in the regulation of the blood-testis-barrier (BTB), tight junction components, and Sertoli cell polarity was suggested by several authors (45, 47, 109). While deletion of *Cdc42* expression in Sertoli cells in the Mori study did not lead to major changes in the BTB integrity and cell polarity, it led to the depletion of the growth factor glial cell line-derived neurotrophic factor (GDNF), a major determinant of spermatogonial proliferation, possibly through the downregulation of canonical PAK1 activity downstream of CDC42 (44).

## Epigenetic Regulators of the Niche

One of the first discovered epigenetic regulators of the SSC niche was the Switch-insensitive 3a (SIN3A) co-repressor protein, part of a massive transcriptional complex that interacts with a wide array of epigenetic regulators (114). The SIN3A transcriptional corepressor complex plays a role in diverse cellular processes such as proliferation, differentiation, tumorigenesis, apoptosis and cell fate determination (113). The classical mechanism of action of this complex is transcriptional silencing through histone deacetylation mediated by HDAC1/2. In the mouse testis, Sertoli cell specific *Sin3a* deletion resulted in a decrease of undifferentiated spermatogonia after birth. The Sertoli cell markers *Kit Ligand (KITL)* and *Gdnf*, which support germ cell proliferation, were not diminished. However, chemokine signaling molecules such as CXCL12/SDF1 and CXCR4, expressed by Sertoli cells and germ cells, respectively, were not detected. This again demonstrates that regulators of germ cell movement toward the periphery of testicular cords and the basement membrane after birth are critical for the establishment of the initial postnatal niche. However, the relationship between SIN3A and the signaling networks governed by GATA4 and ETV5 in Sertoli cells are not yet known.

In 2013, Wu and colleagues identified ARID4A and ARID4B (AT-rich interactive domain-containing protein 4A/B) as additional master regulators critical for the establishment of the niche, in particular during the pro-spermatogonia to SSC transition phase (35, 36). Interestingly, ARID4B is a subunit of the SIN3A transcriptional repressor complex. Sertoli cell ablation of *Arid4B* expression resulted in Sertoli cell detachment from the basement membrane, which precluded niche formation and the movement of pro-spermatogonia toward the periphery of the testicular cords. Without niche support, the germ cells underwent apoptosis. The authors also showed that ARID4B can function as a transcriptional coactivator for androgen receptor (AR) and identified reproductive homeobox 5 (*Rhox5*) (124) as the target gene critical for spermatogenesis (34).

Another epigenetic regulator of the niche is WTAP, or Wilms Tumor 1-associated protein (33). WTAP regulates transcription and translation of genes by depositing N^6^-methyladenosine (m^6^A) marks directly on RNA transcripts or indirectly on transcriptional regulators (125). Jia and colleagues demonstrated that conditional deletion of *Wtap* in mouse Sertoli cells modified pre-mRNA splicing, diminished RNA export and translation, and therefore altered the transcription and translation of many Sertoli cell genes normally marked by m^6^A modification. Many of these genes were critical for SSC maintenance, spermatogonial differentiation, retinol metabolism, and the cell cycle, including *Inhbb*, *Wt1*, *Arid4a*, *Arid4b*, *Etv5*, *Ar*, *Dmrt1*, and *Sin3a* (**Table 1**) (23, 27, 35, 60, 83, 114, 126, 127). Consequently, progressive loss of undifferentiated spermatogonia was observed in WTAP-deficient testes and mice were sterile. Interestingly, while not normally marked by m^6^A modification, *Gdnf*, which is required for SSC maintenance and self-renewal, was also downregulated. The authors surmised that several of the key transcription regulators that have been reported to be important for *Gdnf* transcription contained m^6^A sites and were dysregulated by *Wtap* knockout.

## Single Cell RNA-Seq and Spatial Transcriptional Dissection of Perinatal and mature Sertoli Cells

Single cell characterization of developing and mature Sertoli cells in rodents and humans, as well as their spatial transcriptional dissection, uncovered many genes potentially important for the organization of the niche, and are providing a large resource for functional analysis of possible signaling pathway networks (102, 128–132). All studies demonstrated that mouse Sertoli cells undergo stepwise changes during the perinatal period, which are dependent on the expression of *SOX9, AMH, GATA1-4, DMRT1, NR3C1* and their target genes (**Table 1**) (32, 58, 101, 102, 116). Notably, as predicted, expression of cell cycle genes decreases as Sertoli cells mature after birth. Further, these data demonstrated a postnatal increase in expression of Sertoli-Sertoli cell junctions and germ cell-Sertoli cell junction signaling (102). Zhao and colleagues identified three stages of postnatal Sertoli cells maturation in humans. In stage a (2-5 years old), the top three differentially expressed genes were *EGR3*, *JUN*, and *NR4A1* (**Table 1**) (30, 86). In stage b (8-11 years) *S100A13*, *ENO1*, and *BEX1* were prominently expressed, while in stage c (17 years to adult) *HOPX, DEFB119*, and* CST9L* were upregulated (**Table 1**) (49, 57, 81). Gene Ontology and Ingenuity Pathway Analysis (IPA) at each of the three stages indicated that genes ensuring proliferation and maintenance of cell numbers were prominently expressed in stage a (EGF, IGF, and ERK5 signaling), RHOA/ACTB motility and remodeling of Sertoli-Sertoli epithelial junctions were a feature of stage b, and pathways of cholesterol biosynthesis and germ cell-Sertoli cell junction signaling were increased in stage c (59, 82). In addition, protein transmembrane transport, phagosome maturation, and cellular metabolic processes were upregulated in stage c, confirming that the most important functions of mature Sertoli cells are the production of growth factors, phagocytosis of germ cells and metabolites processing. Collectively, these data indicate that single cell RNA-seq and spatial transcriptomic characterization of Sertoli cells generate reliable resources for future mechanistic studies of master regulators of the niche and their targets at different time points.

## Sertoli Cell Factors Controlling SSC Maintenance And Self-Renewal.

In the seminiferous epithelium, Sertoli cells produce a number of soluble factors that are under the control of the above-described master regulators. These growth factors are critical for pro-spermatogonial maintenance in the fetus, maintenance of the SSC pool, self-renewal of SSCs after birth, and the onset of germ cell differentiation. The most critical factors include glial cell line-derived neurotrophic factor (GDNF) (75), colony-stimulating factor 1 (CSF1) (12), fibroblast growth factor 2 (FGF2) (65, 66), leukemia inhibitory factor (LIF) (10) and WNT family proteins (50, 122). They all bind to their cognate receptors at the surface of SSCs or undifferentiated spermatogonia and activate the MAPK or PI3K/AKT pathway to drive the cell cycle. They also promote SSC proliferation *in vitro*, which can be demonstrated by increased testes colonization after transplantation. KITL, the ligand for KIT receptor, and retinoic acid (RA) are considered major determinants of germ cell differentiation after birth, promote the switch between undifferentiated and differentiating spermatogonia and trigger meiotic entry (94, 133, 134).

### Glial Cell Line-Derived Neurotrophic Factor

GDNF is a member of the transforming growth factor beta (TGF-b) superfamily that binds to the GFRA1/RET receptor complex at the surface of SSCs, A_paired_ and some A_aligned_ spermatogonia (75, 77). Meng and colleagues were first to demonstrate that GDNF haploinsufficiency in mice induced fertility defects after birth (75). The mice were fertile but exhibited increased numbers of seminiferous tubules lacking spermatogonia as they aged. In addition, transgenic animals overexpressing *Gdnf* accumulated undifferentiated spermatogonia. In 2006, Naughton and colleagues disrupted the expression of *Ret* and *Gfra1* at the surface of SSCs, which resulted in their loss and led to the definitive proof of the critical function of this receptor-ligand interaction (78). Together with FGF2 and LIF, GDNF is critical for the self-renewal of SSCs in short- and long-term cultures (66). Because of its importance for spermatogenesis, efforts were made to understand the temporal regulation of its expression. Low levels of GDNF and RET are already present in the fetal gonad (76, 110). Since pro-spermatogonia do not proliferate until after birth, GDNF is therefore only necessary for their maintenance, highlighting the importance of its dosage (98). GDNF expression then increases until it reaches a peak at days 3-7 after birth (110, 135, 136). One interesting feature of GDNF expression in the adult is its cyclic pattern throughout the stages of the seminiferous epithelium. Cyclical production of soluble factors according to stages was demonstrated earlier by Johnston and colleagues using transillumination-assisted microdissection and microarray analysis (137). In the rat, GDNF expression is highest at stages XIII-I, and lowest at stage VII of the seminiferous epithelium (138), while in the mouse its expression is highest at stages IX-I and lowest at stages V-VIII when most cells are quiescent and the majority of A_aligned_ spermatogonia transition to the differentiating A1-A4 cells (85, 98, 139). When GDNF was ectopically overexpressed by Sertoli cells in Stages V-VIII, the number of GFRA1+/LIN28^-^ germ cells, a subtype of A_s_ spermatogonia with enhanced self-renewal capacity, was increased (97, 98).

**Figure 2:**
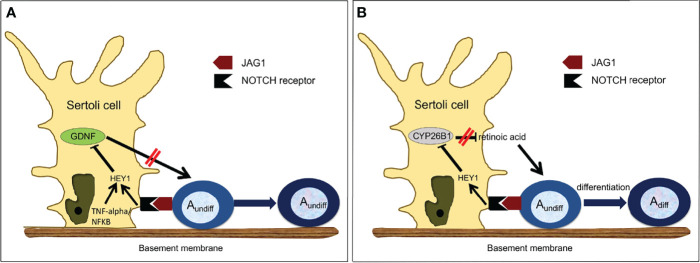
Proposed Model of Regulation of Germ Cell Homeostasis by NOTCH Signaling. **(A)** Regulation of GDNF expression in Sertoli cells. GDNF is produced by Sertoli cells and normally increases Asingle, Apaired and some Aligned spermatogonia proliferation. However, as the number of undifferentiated spermatogonia increases, more JAG1 ligand is available to activate NOTCH signaling in Sertoli cells. Activated NOTCH will down-regulate the expression of GDNF through HES/HEY, which will decrease the number of undifferentiated spermatogonia, re-establishing GDNF production. Inhibition of GDNF by HES/HEY can be potentiated by the TNF-alpha/NF-KappaB pathway. **(B)** Regulation of CYP26B1 expression in Sertoli cells. CYP2681 is produced by Sertoli cells and normally degrades retinoic acid. However, as the number of undifferentiated spermatogonia increases, in particular Aaligned spermatogonia, more JAG1 ligand is available to activate NOTCH signaling in Sertoli cells. Activated NOTCH will down-regulate the expression of CYP26B1, which a llows retinoic acid to trigger the transition from undifferentiated to differentiating spermatogonia.

Several mechanisms regulating GDNF expression have been recently proposed. Garcia and colleagues established Sertoli cell-specific gain-of-function and loss-of-function mouse models of NOTCH receptor signaling (80, 100). Constitutive activation of this pathway in Sertoli cells led to a complete lack of germ cells by P2, and infertility. Expression of GDNF by Sertoli cells was significantly downregulated in the perinatal and adult testis and was due to upregulation of *Hes/Hey* transcription factors, which are canonical NOTCH targets and transcriptional repressors that bind to the GDNF promoter (80, 85). Further, loss-of-function of *Rbpj*, a mediator of NOTCH, and downregulation of *Hes/Hey*, led to upregulation of *Gdnf* expression (80) (**Table 1**). Importantly, the NOTCH ligand JAG1 was expressed mainly by undifferentiated spermatogonia (85). Consequently, the accumulation of undifferentiated spermatogonia around stage VII might increase NOTCH activity in Sertoli cells through JAG1, triggering the observed increase of Hes/Hey inhibitors at this stage and decrease in GDNF expression, leading to its cyclic expression. Therefore, spermatogonia, when in sufficient numbers, regulate their own homeostasis through downregulation of GDNF (55). These data are consistent with the observation that in wild type mice, the absence of germ cells triggered by busulfan treatment correlated with higher expression of GDNF (85, 135, 140) (**Figure 2A**).

Other interesting mechanisms of GDNF regulation have been recently proposed. Given the fact that retinoic acid (RA) concentration is high when GDNF is low during the cycles of the seminiferous epithelium (141), Saracino and colleagues tested whether RA was a direct inhibitor of GDNF expression (142). Using *ex vivo* cultured immature testes and staged adult seminiferous tubules, they showed that negative regulation of *Gdnf* by RA indeed takes place in these models and demonstrated that *Gdnf* expression is directly regulated by RA through a mechanism involving a RARE-DR5 binding site on the *Gdnf* promoter. Negative regulation requires retinoic acid receptor (RARα) and induces a strong decrease of histone H4 acetylation levels around the transcription start. Further, because of the existence of a NF-kappaB binding site in the GDNF promoter, the same group investigated how TNF-alpha might influence GDNF expression (99). They demonstrated that in primary Sertoli cells, TNF-alpha induces the expression of the transcriptional repressor *Hes1* by a NF-KappaB-dependent mechanism, which in turn downregulates GDNF. Therefore, TNF-alpha and NOTCH signaling may converge to regulate the expression of *Hes1* and its target genes, including GDNF (**Figure 2A**).

### Fibroblast Growth Factor *(FGF2)*

While GDNF is a critical component of the niche, many *in vivo* and *in vitro* experiments demonstrated that other factors are needed to support maintenance and self-renewal of SSCs. Earlier examination of perinatal Sertoli cells demonstrated that they expressed FGF2, and that this expression was stimulated by follicle-stimulating hormone *in vitro* (FSH) (64). Together with EGF, LIF, and GDNF, fibroblast growth factor (FGF2) has been used to sustain the long-term proliferation of SSCs in culture (66, 143). Further, Takashima and colleagues demonstrated that FGF2 could induce SSC self-renewal alone in culture through activation of the transcription factors ETV5 and BCL6B (**Table 1**) (37, 38, 60, 62, 63, 67). They also showed that FGF2-depleted mouse testes produced increased levels of GDNF, which correlated with SSCs enrichment. This suggests that a balance or complementation between FGF2 and GDNF exists to maintain the stem cell pool (67). More recently, additional studies comparing the effects of GDNF and FGF2 on the proliferation of undifferentiated spermatogonia demonstrated that while both factors expanded the GFRA1+ population, FGF2 rather expanded a subpopulation of cells expressing RARG, which were therefore more susceptible to differentiate (68). This emphasizes the complex nature of signaling and a growth factor demand that is modulated upon the need to maintain germ cell homeostasis.

### Other Growth Factors

Platelet-derived growth factor (PDGF) is specifically produced by Sertoli cells. In rodents, PDGF is critical for prospermatogonia proliferation after birth (103, 104) and cooperates with estrogen signaling (106). Exposure to xenoestrogens in the environment might disrupt crosstalk between PDGF and estrogen-driven signaling pathways. This could lead to alteration of prospermatogonia behavior and induce preneoplastic states (105). Vascular endothelial growth factor A (VEGFA) family members and their receptors are all produced by germ cells, Sertoli, cells and interstitial cells (117, 118). However, only the pro-angiogenic isoform VEGFA164 promotes SSC self-renewal, as determined by the SSC transplantation assay (119). WNT signaling plays a role in SSC maintenance (50, 144). WNT5A is produced by Sertoli cells but does not induce self-renewal. It rather promotes SSCs survival through a β-catenin (CTNNB1)-independent mechanism that activates mitogen-activated protein kinase 8 (MAPK8 or JNK) (50). Confirming this data, CTNNB1 ablation in germ cells led to spermatogenesis disruption but not to SSC loss (51, 52). Finally, leukemia inhibitory factor (LIF) has been used for decades to maintain undifferentiated embryonic stem cells *in vitro*, therefore an investigation of its expression in Sertoli cells and its effects on SSCs, at least *in vitro*, was attempted early on (96). LIF production in Sertoli cells was shown to depend on tumor necrosis factor (TNFα) (96) and is still widely used in cultures of primordial germ cells, pro-spermatogonia, and SSCs of many different species. However, LIF does not induce SSC self-renewal, and is rather used to maintain survival and start long-term SSC cultures (10).

## Sertoli Cell Factors Controlling Spermatogonial Differentiation

### Regulation of KIT/KITL

Activation of the KIT tyrosine kinase receptor by its ligand KITL is required for the survival and proliferation of primordial germ cells (PGCs) (90). KIT is downregulated in pro-spermatogonia, which stop proliferating once they enter the fetal gonads. After birth, KIT is re-expressed in differentiating spermatogonia (87, 88), which proliferate under the influence of KIT ligand (KITL) produced by Sertoli cells. Together with retinoic acid (RA), the KIT/KITL system is important for triggering meiotic entry of type B spermatogonia (92, 93), and KITL has been recently used in culture to differentiate rat spermatogonia without serum or somatic cells (95). Because KIT/KITL signaling is important not only for germ cells, but also for haematopoietic stem cell and melanoblasts, mechanisms controlling KIT transcription have been extensively studied. Further, KIT is mutated in about 25% of seminoma (91), and accounts for secondary mutations that confer resistance to drugs in other cancers. Therefore, regulation of its expression and identification of downstream effectors as druggable targets are of particular interest. Earlier studies have demonstrated that KIT expression in undifferentiated spermatogonia is repressed by PLZF (promyelocytic leukemia zinc finger), which is a transcriptional repressor with local and long-range chromatin remodeling activity (107, 108). Further, Dann and colleagues demonstrated that RA triggered spermatogonial differentiation through downregulation of PLZF (145). Thus, one mechanism by which PLZF maintains the pool of spermatogonial stem cells is through a direct repression of *Kit* transcription. The main mechanism of KIT upregulation involves the helix-loop-helix transcription factors SOHLH1 and SOHLH2 (Spermatogenesis and Oogenesis HLH1/2). Both factors are expressed in differentiating spermatogonia and their deletion leads to the disappearance of KIT-expressing spermatogonia. Further, ChIP-PCR analysis demonstrated that SOHLH1 binds the *Kit* promoter to activate its transcription (115). While investigations have mostly focused on the regulation of KIT, few studies have explored the regulation of KITL expression in the past 10 years. However, one study by Correia and colleagues demonstrated that 100 nM estrogen induced a decrease in *Kit* expression while increasing expression of *Kitl* in adult rat seminiferous tubules cultured ex vivo (89). Altered expression of the KIT/KITL system decreased germ cell proliferation and promoted apoptosis, which is not in accord with the data of previous studies (146).

### Regulation of Retinoic Acid Activity

Rats and mice deprived of dietary retinoic acid (RA) can only produce A_undiff_ spermatogonia and are sterile (147, 148). Since these earlier studies, it has been well documented that retinoic acid (RA) activity is a major determinant of the transition between undifferentiated and differentiating germ cells, and that RA also drives the meiotic process and spermatid maturation at stage VIII of the seminiferous epithelium (134, 149). It has been proposed that pulses of RA are triggered around this stage by somatic cells and germ cells to allow proper germ cell differentiation and maturation (150). This implies that RA must be degraded during the other stages. Recently, Parekh and colleagues demonstrated an inverse relationship between the expression of *cytochrome P450 family 26 subfamily B member 1 (Cyp26b1)*, an enzyme that degrades RA (54), and NOTCH activity in Sertoli cells (56). They further provided evidence that in the adult testis activated NOTCH signaling in Sertoli cells down-regulates *Cyp26b1* expression through the HES/HEY transcriptional repressors that bind to the *Cyp26b1* promoter (56). Importantly, expression of these inhibitors is highest at stage VIII of the seminiferous epithelium (85). They also demonstrated that A_aligned_ spermatogonia, through their expression of the NOTCH receptor JAG1, were activating the NOTCH/HES/HEY axis in Sertoli cells and were responsible for *Cyp26b1* down-regulation at stage VIII, allowing RA activity and therefore triggering their own differentiation into A_1_ spermatogonia (**Figure 2B**).

## Conclusion

The Sertoli cell orchestrates spermatogenesis and is a major component of the SSC niche. The past decade has seen an increase in our understanding of these processes at the molecular level. In the perinatal testis, Sertoli cells support multiple aspects of germ cell development through paracrine factors, but the master regulators of the niche and the signaling networks regulating these soluble factors have just begun to be identified. State-of-the-art technologies exist that should help dissect the functions of novel genes and signaling pathways in Sertoli cells in the future. The efforts that were spent understanding the cyclic regulation of GDNF and Cyp26b1, and by extension RA, should be expanded to other growth and differentiation factors. In particular, surprisingly little is known about the signals that germ cells send to Sertoli cells and their neighboring germ cells. We hope that the use of spatial transcriptomics will help uncover the molecular signals and pathways that germ cells and Sertoli cells use to communicate between each other to direct testis function and maintain homeostasis. We have highlighted JAG1/NOTCH signaling as one possible mechanism that fulfills this role, but other modes of germ cell to Sertoli cell communication exist that still need to be identified.

## Author Contributions

All authors listed have made a substantial, direct, and intellectual contribution to the work and approved it for publication.

## Funding

This work was funded by NIH R01 HD081244 and NIH R03 HD101650.

## Conflict of Interest

The authors declare that the research was conducted in the absence of any commercial or financial relationships that could be construed as a potential conflict of interest.

## Publisher’s Note

All claims expressed in this article are solely those of the authors and do not necessarily represent those of their affiliated organizations, or those of the publisher, the editors and the reviewers. Any product that may be evaluated in this article, or claim that may be made by its manufacturer, is not guaranteed or endorsed by the publisher.
